# Medical students and physical education students as CPR instructors: an appropriate solution to the CPR-instructor shortage in secondary schools?

**DOI:** 10.1007/s12471-016-0838-2

**Published:** 2016-05-18

**Authors:** P. J. P. M. Cuijpers, G. Bookelman, W. Kicken, W. de Vries, A. P. M. Gorgels

**Affiliations:** CAPHRI School for Public Health and Primary Care, Maastricht University, PO Box 616, 6200 MD Maastricht, The Netherlands; department Cardiology, Maastricht University MedicalCenter +, Maastricht, The Netherlands; Welten Institute, Research Centre for Learning, Teaching and Technology, The Open University of The Netherlands, Heerlen, The Netherlands; Knowledge Centre ACM Education, Elburg, The Netherlands

**Keywords:** Cardiopulmonary resuscitation, CPR training, CPR-instructor qualification, Secondary school students

## Abstract

**Background:**

Integrating cardiopulmonary resuscitation (CPR) training in secondary schools will increase the number of potential CPR providers. However, currently too few certified instructors are available for this purpose. Training medical students and physical education student teachers to become CPR instructors could decrease this shortage.

**Aim:**

Examine whether medical students and physical education student teachers can provide CPR training for secondary school pupils as well as (i. e., non-inferior to) registered nurses.

**Methods:**

A total of 144 secondary school pupils were randomly assigned to CPR training by a registered nurse (*n* = 12), a  medical student (*n* = 17) or a physical education student teacher (*n* = 15). CPR performance was assessed after training and after eight weeks in a simulated cardiac arrest scenario on a resuscitation manikin, using manikin software and video recordings.

**Results:**

No significant differences were found between the groups on the overall Cardiff Test scores and the correctness of the CPR techniques during the post-training and retention test. All pupils showed sufficient CPR competence, even after eight weeks.

**Conclusion:**

Training by medical students or physical education student teachers is non-inferior to training by a registered nurse, suggesting that school teachers, student teachers and medical students can be recruited for CPR training in secondary schools.

## Introduction

By increasing the number of citizens trained to perform basic cardiopulmonary resuscitation (CPR), outcomes of out-of-hospital cardiac arrest (OHCA) could be improved [1–4]. Over the last years, also in the Netherlands, efforts have been spent to improve survival for this important healthcare problem, which have already succeeded in improving outcome [5–8]. A promising strategy to increase the number of trained citizens is to implement CPR training in secondary schools [9–13]. However, at present too few certified instructors are available for this purpose [14–16]. In the Netherlands, according to the requirements of the Dutch Resuscitation Council, only physicians, registered nurses, paramedics and qualified first aid instructors are allowed to provide CPR training [17]. Training medical students and physical education student teachers to become CPR instructors could decrease this shortage [18–22]. The aim of this study was to examine whether medical students and physical education student teachers can provide CPR training for secondary school pupils as well as (i. e., non-inferior to) registered nurses.

## Methods

### Study setting

This study is a practical randomised non-inferiority study. It had to be executed within the regular school program, also taking into account the availability of registered nurses, physical education student teachers and medical students. In total 44 CPR instructors participated in the study. Tab. 1 provides an overview of the characteristics of the CPR instructors. The registered nurses were employed at local hospitals and had practical CPR experience in hospital settings. The physical education student teachers were in the second year of their training and had no experience with CPR. The medical students were in the second or third year of medical school and also had no experience with CPR.

**Tab. 1:** Demographic data CPR instructors

	RN	PE	MS	*p*
Number	12	15	17	
Gender (male/female)	5/7	11/4	4/13	0.018
Age (years) (mean)	29.2	22.3	21.0	0.000
Age (years) 95 % CI	25.6–32.9	21.1–23.5	20.4–21.7	

*RN* registered nurses, *PE* physical education student teachers, *MS* medical students

Pupils were recruited from two secondary schools, participating in the Heart Beat Survival Programme, an initiative in the Dutch province of Limburg to study the implementation of CPR training in secondary schools. A total of 144 pupils (82 boys, 62 girls) aged 13 to 16 (mean age 14.4, SD 0.6) participated in the study. Pupils were randomly assigned to one of three CPR training groups which were led by either a registered nurse, a physical education student teacher or by a medical student. Tab. 2 provides an overview of the characteristics of the participants per instructor group. The school management gave consent for the participation of their pupils in this study and the pupils’ parents were informed by letter.

**Tab. 2:** Demographic data secondary school pupils

		RN group	PE group	MS group	*p*
Number of pupils		41	50	53	
Gender (male/female)		20/21	28/22	34/19	0.324
Age (years)	13 14 15 16	1 25 14 1	1 29 19 1	1 34 16 2	0.987
Educational level	HAVO^a^ VWO^b^	24 17	19 31	25 28	0.149

*RN* registered nurses, *PE* physical education student teachers, *MS* medical students

^a^ *HAVO* Dutch upper general secondary education

^b^ *VWO* Dutch pre-university education

### Instructor training

All instructors were trained by certified instructor trainers of the Dutch Resuscitation Council (DRC) according to the 2005 CPR guidelines of the European Resuscitation Council (ERC). All instructors were informed about the design of the study and gave consent to participate.

### CPR training at school

**Fig. 1:**
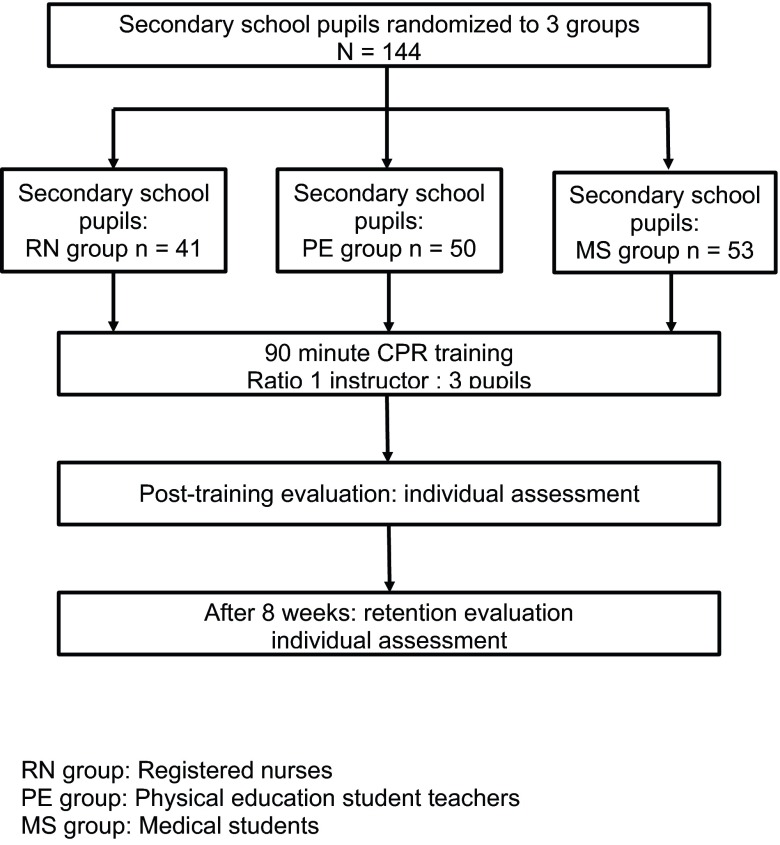
Outline of study design and participant flow

All pupils were trained during two subsequent months. Training was scheduled during two successive regular physical education lessons. After the CPR training, pupils’ CPR skills were individually assessed. Eight weeks after the CPR training, the pupils’ skills were retested. This retention assessment was identical to the post-training assessment. Fig. 1 provides an overview of the procedure of the study.

The CPR training was based on the standard ERC protocol with a small adjustment of the prescribed instructor-trainee ratio of 1:6 and training duration of four hours to match the practical groups in the schools. Each instructor trained three pupils for 90 minutes (i. e. two lessons) in the gymnasium of the school. The instructor started with an introduction followed by a demonstration of CPR on a resuscitation manikin Skill reporter ResusciAnne and the Laerdal PC Skill Reporting System version 2.0 (Laerdal Medical, Stavanger, Norway). To demonstrate the application of an Automatic External Defibrillator (AED) an AED trainer (Lifepak CR Plus training device Medtronic Physio Control Corp., Redmond, USA) was used.

### CPR and AED skills assessment

The CPR skills and the use of an AED were assessed directly after the training and eight weeks later in a separate session. Pupils were informed that their CPR skills would be individually assessed and that their performance would be videotaped. The assessment started as soon as pupils entered the classroom. A manikin was lying on the floor and a ‘bystander’ (i. e., an instructor) was in the classroom. An AED-trainer device was also present in the room and was programmed for a two-shock scenario. The bystander was instructed to tell the pupil that he did not know how to perform CPR or how to use an AED adequately. The assessment and recording were stopped either after the pupil recommenced CPR after providing the second shock with the AED trainer device or two minutes after the pupil had not activated the AED trainer device.

The performance was scored separately after the assessment session using the Cardiff list and the videotape. To assess the pupils’ CPR and AED skills a form was composed based on the Cardiff Test [23]. Only the Cardiff Test items relevant for correct application of the ERC guidelines (2005) were used: (a) approach section, (b) CPR sequence and (c) AED sequence. Pupils could attain a maximum score of 74 points. A total score of 60 points or more (i. e., ≥80 % correct) was considered to represent well acquired CPR skills. The adequate application of specific techniques or technical skills during CPR such as ventilation volume, compression depth and rate and compression/ventilation ratio was automatically registered by the manikin software. Assessment was done by experienced instructors blinded for the study group. Videos were taken against a neutral background to ensure that assessors were blinded for the study group.

### Statistical analysis

Statistical calculations were performed using the SPSS 19.0 package (SPSS Inc. Chicago II, USA). Statistical significance was accepted at a two-sided *p*‑value of <0.05 or when confidence intervals did not include unity. In addition, Kruskal-Wallis analysis and post hoc Bonferroni analyses were computed to identify the differences between instructor groups.

## Results

### Post-training CPR skills

#### CPR approach, CPR and AED sequence.

Tab. 3 shows an overview of the assessment scores and of the performance of the technical skills during the CPR of the pupils in the three groups, both of the assessment immediate post-training and after eight weeks. Pupils scored a mean of 67.0 points (*SD =* 3.8) on the Cardiff Test items regarding CPR approach and sequence. In the medical student group, a larger number of pupils gained high assessment scores compared with the other groups, but this difference was not significant. Regarding the overall scores, the Kruskal-Wallis analysis showed no significant differences (*χ*^2^ = 1.58, *p* = 0.45) between pupils in the registered nurse group (*M* = 66.55, *SD* = 3.99), the physical education student teacher group (*M* = 66.50, *SD* = 3.46) or the medical student group (*M* = 67.65, *SD =* 4.06). Examining the CPR skills at a more specific level (i. e. per Cardiff Test item), pupils in the registered nurse group were found to perform better at ‘safe approach’ (*χ*^2^ = 6.74, *p* = 0.03), than pupils in the other groups. Pupils in the medical student group performed better at ‘call 112’ (*χ*^2^ = 13.42, *p* = 0.001) and ‘placing defibrillator pads’ (*χ*^2^ = 13.80, *p* = 0.001), than pupils in the other groups.

**Tab. 3:** Results of post training test and retention test evaluation

	RN group		PE group		MS group		Total		
	*n* = 41		*n* = 50		*n* = 53		*n* = 144		
	Mean ± SD	95 % CI	Mean ± SD	95 % CI	Mean ± SD	95 % CI	Mean ± SD	95 % CI	*P* ^*^
*Approach sequence*									
Post training	18.9 ± 2.4	18.1–19.6	18.6 ± 2.9	17.8–19.4	19.9 ± 2.0	19.3–20.5	19.2 ± 2.5	18.8–19.6	0.020
Retention test	18.5 ± 2.6	17.7–19.4	18.3 ± 2.9	17.4–19.1	19.0 ± 2.9	18.1–19.8	18.6 ± 2.8	18.1–19.1	0.454
*CPR sequence*									
Post training	26.4 ± 2.5	25.7–27.2	26.1 ± 2.5	25.4–26.8	26.7 ± 2.5	26.0–27.4	26.4 ± 2.5	26.0–26.8	0.497
Retention test	25.5 ± 3.5	24.4–26.6	25.5 ± 3.1	24.6–26.4	26.3 ± 2.5	25.6–27.0	25.8 ± 3.0	25.3–26.3	0.290
*AED sequence*									
Post training	20.9 ± 1.3	20.5–21.4	21.4 ± 1.2	21.1–21.8	21.8 ± 0.9	21.5–22.0	21.4 ± 1.2	21.2–21.6	0.002
Retention test	20.7 ± 1.2	20.3–21.1	20.8 ± 1.3	20.5–21.2	21.6 ± 0.8	21.4–21.9	21.1 ± 1.2	20.9–21.3	<0.001
*Total sequence*									
Post training	66.2 ± 3.9	65.0–67.5	66.1 ± 3.3	65.2–67.1	68.3 ± 3.7	67.4–69.4	67.0 ± 3.8	66.4–67.6	0.003
Retention test	64.8 ± 5.4	63.1–66.5	64.6 ± 4.8	63.2–66.0	66.9 ± 3.7	65.9–68.0	65.5 ± 4.7	64.7–66.3	0.021
*Compression frequence*									
Post training	95.0 ± 11.7	91.3–98.7	100.5 ± 16.1	95.9–105.1	98.1 ± 12.0	94.8–101.4	98.1 ± 13.6	95.8–100.3	0.161
Retention test	99.6 ± 14.7	95.0–104.2	104.8 ± 15.4	100.4–109.2	100.0 ± 14.9	95.9–104.1	101.6 ± 15.1	99.1–104.0	0.165
*Compression depth*									
Post training	34.7 ± 9.1	31.8–37.6	35.9 ± 7.0	33.9–37.9	38.2 ± 8.4	35.9–40.6	36.4 ± 8.2	35.0–37.8	0.098
Retention test	35.1 ± 10.4	31.8–38.4	37.2 ± 7.9	35.0–39.5	36.4 ± 9.3	33.9–39.0	36.3 ± 9.1	34.8–37.8	0.532
*Ventilation volume*									
Post training	480 ± 187	421–539	503 ± 230	438–568	496 ± 214	437–555	494 ± 211	459–529	0.872
Retention test	523 ± 263	440–606	502 ± 317	412–592	550 ± 248	481–618	526 ± 277	479–571	0.686

*CPR* cardiopulmonary resuscitation, *AED* automatic external defibrillator *RN* registered nurse, *PE* physical education teacher, *MS* medical student ^*^significance level *p* < 0.05

#### CPR technical skills.

The Kruskal-Wallis analysis showed no significant differences regarding correctness of the inflation volume (*χ*^2^ = 1.59, *p* = 0.45), compression depth (*χ*^2^ = 3.99, *p* = 0.13) or compression rate (*χ*^2^ = 3.14, *p* = 0.20).

### Retention CPR skills

#### CPR approach, CPR sequence and AED sequence.

Eight weeks after the CPR training the overall performance score of all pupils decreased slightly to 65.5 (SD = 4.7). However, this score still represents well-developed CPR skills (i. e., ≥60 points). In addition, significant differences were found between the overall performance of pupils in the three groups (*χ*^2^ = 7.27, *p* = 0.03): Pupils in the medical student group had slightly higher scores (*M* = 66.9, *SD =* 3.7) than pupils in the registered nurse group (*M* = 64.8, *SD* = 5.4) and pupils in the physical education student teacher group (*M* = 64.6, *SD* = 4.8). An analysis with respect to specific skills (i. e., items of the Cardiff Test) showed that pupils in the medical student group nscored significantly higher at ‘hand position area’ (*χ*^2^ = 15.76, *p* = 0.001) and ‘placing defibrillator pads’ (*χ*^2^ = 18.23, *p* = 0.001) than pupils in the other groups.

#### CPR technical skills.

No significant differences were seen between pupils in the three groups with respect to correctness of the inflation volume (*χ*^2^ = 1.25, *p* = 0.54), compression depth (*χ*^2^ = 1.02, *p* = 0.60) or compression rate (*χ*^2^ = 3.69, *p* = 0.16).

**Fig. 2:**
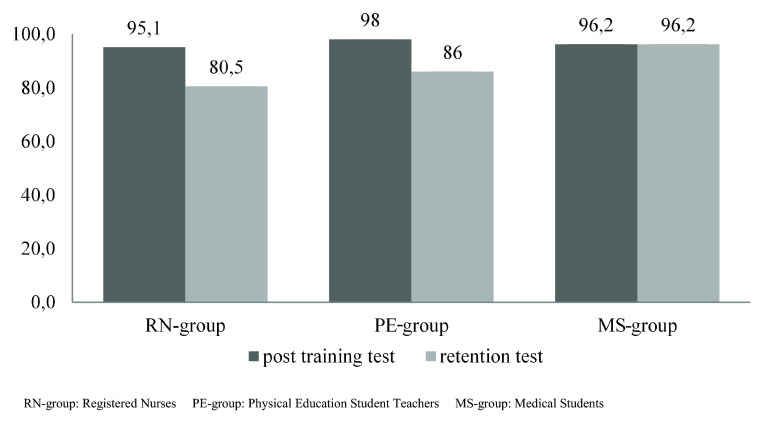
Provides the percentages of pupils passing according to the Cardiff test score.

## Discussion

Our results support the hypothesis that secondary school pupils who are trained by physical education student teachers or medical students perform non-inferiorly to pupils trained by registered nurses, both immediately after the training and after eight weeks. Medical students even appeared to have slightly but significantly higher overall scores for three Cardiff Test items (i. e., CPR approach, CPR sequence and AED sequence) eight weeks after the training. Slight differences regarding specific items of the Cardiff Test existed between the medical student and registered nurse group. Pupils trained by registered nurses performed better at ‘safe approach’, whereas those trained by medical students at ‘phone 112’ and ‘placing defibrillator pads’. This could be due to the in-hospital experience of the registered nurses. The medical students on the other hand were more inclined to strictly follow the CPR guidelines and likely put emphasis on providing the correct information to the 112 operator and to placing the pads correctly. No differences were found regarding inflation volume, compression depth and compression rate between the groups either at the post-training assessment or at the retention assessment. These results support the idea that also medical students and physical education student teachers could be deployed as CPR instructors to train larger numbers of secondary school pupils.

Our study results confirm that CPR training could be done by medical students, student teachers and thus also school teachers, equally well as by certified instructors such as registered nurses. This situation has several advantages: It is not only less costly, but also overcomes problems such as matching school hours and working hours of registered nurses. It is a long-term investment, as teachers are connected to a school for a prolonged period of time. Also, teachers can repeat the training throughout the pupils school career [24].

In our study CPR skills were trained in one single session of 90 minutes, being the best feasible possibility within the school curriculum [25]. To train CPR skills effectively, repetition is important, requiring the availability of sufficient numbers of instructors [26].

Deploying physical education student teachers to provide CPR training not only helps to reduce the shortage of CPR instructors in the short term, throughout their teacher training, but also on the long term once they are employed as physical education teachers at school.

## Limitations

Due to the practical design of our study a less than envisaged number of registered nurses participated, causing a disproportionate number of study instructors per group. However, this restricted availability reflects the real world situation and also accounts for physicians and resuscitation training officers. Also, data were analysed using non-parametric analyses, students were randomly assigned to groups, were of comparable age and received training under the same standardised conditions, training and assessment were in line with the ERC guidelines, the validated Cardiff Test was used to score performance lists and quantitative performance was monitored through qualitative manikins.

As study endpoints only short-term results were considered. Therefore it is not known whether the conclusions reached would be similar at longer follow-up. This study was executed in the first month of 2010, following the guidelines that were valid at that time. However, there is no reason to assume the results are not appropriate for training programs using the current guidelines.

## Conclusion

No relevant differences existed between secondary school pupils trained by physical education student teachers or medical students, and pupils trained by registered nurses. A slightly better performance in pupils trained by medical students was observed, which remained after eight weeks.

This suggests that student instructors such as physical education student teachers and medical students are as competent as registered nurses to train CPR at secondary schools. This may have important implications regarding the implementation of CPR training in secondary schools: if these student instructors are accredited as such, more secondary school pupils can be trained, given the restricted availability of the classical instructors. This will increase the number of CPR-instructed civilians in our society, which in turn may provide a better outcome after OHCA.

## References

[1] Soar J, Mancini ME, Bhanji F (2010). Part 12: Education, implementation, and teams: 2010 International Consensus on Cardiopulmonary Resuscitation and Emergency Cardiovascular Care Science with Treatment Recommendations. Resuscitation.

[2] Lindner TW, Søreide E, Nilsen OB, Torunn MW, Lossius HM (2011). Good outcome in every fourth resuscitation attempt is achievable – an Utstein template report from the Stavanger region. Resuscitation.

[3] Søreide E, Morrison L, Hillman K (2013). The formula for survival in resuscitation. Resuscitation.

[4] van der Wall EE (2003). Out-of-hospital arrest: from innocent bystander to involved citizen responder. Neth Heart J.

[5] Berdowski J, Blom MT, Bardai A, Tan HL, Tijssen JGP, Koster RW (2011). Impact of onsite or dispatched automated external defibrillator use on survival after out-of-hospital cardiac arrest. Circulation.

[6] Blom MT, Beesems SG, Homma PCM (2014). Improved survival after out-of-hospital cardiac arrest and use of automated external defibrillators. Circulation.

[7] Boyce LW, Vliet Vlieland TP (2015). High survival rate of 43 % in out-of-hospital cardiac arrest patients in an optimised chain of survival. Neth Heart J.

[8] Ferreira I, Schutte M, Oosterloo E (2009). Therapeutic mild hypothermia improves outcome after out-of-hospital cardiac arrest. Neth Heart J.

[9] Lockey AS, Georgiou M (2013). Children can save lives. Resuscitation.

[10] Cave DM, Aufderheide TP, Beeson J (2011). Importance and implementation of training in cardiopulmonary resuscitation and automated external defibrillation in schools: a science advisory from the American Heart Association. Circulation.

[11] Reder S, Cummings P, Quan L (2006). Comparison of three instructional methods for teaching cardiopulmonary resuscitation and use of an automatic external defibrillator to high school students. Resuscitation.

[12] Bohn A, Van Aken HK, Möllhoff T (2012). Teaching resuscitation in schools: annual tuition by trained teachers is effective starting at age 10. A four-year prospective cohort study. Resuscitation.

[13] Kanstad BK, Nilsen SA, Fredriksen K (2011). CPR knowledge and attitude to performing bystander CPR among secondary school students in Norway. Resuscitation.

[14] Jiménez-Fábrega X, Escalada-Roig X, Miró O (2009). Comparison between exclusively school teacher-based and mixed school teacher and healthcare provider-based programme on basic cardiopulmonary resuscitation for secondary schools. Emerg Med J.

[15] Mpotos N, Vekeman E, Monsieurs K, Derese A, Valcke M (2013). Knowledge and willingness to teach cardiopulmonary resuscitation: a survey amongst 4273 teachers. Resuscitation.

[16] McCluskey D, Moore P, Campbell S (2010). Topping a. Teaching CPR in secondary education: the opinions of head teachers in one region of the UK. Resuscitation.

[17] De Vries W, Maas M (2008). Opleidingsbeleid instructeurs gebonden onderwijs. Nederlandse Reanimatie Raad.

[18] Iserbyt P, Byra M (2013). The design of instructional tools affects secondary school students’ learning of cardiopulmonary resuscitation (CPR) in reciprocal peer learning: a randomized controlled trial. Resuscitation.

[19] Breckwoldt J, Beetz D, Schnitzer L, Waskow C, Arntz H-R, Weimann J (2007). Medical students teaching basic life support to school children as a required element of medical education: a randomised controlled study comparing three different approaches to fifth year medical training in emergency medicine. Resuscitation.

[20] Gregory A, Walker I, McLaughlin K, Peets AD (2011). Both preparing to teach and teaching positively impact learning outcomes for peer teachers. Med Teach.

[21] Perkins GD, Hulme J, Bion JF (2002). Peer-led resuscitation training for healthcare students: a randomised controlled study. Intensive Care Med.

[22] Toner P, Connolly M, Laverty L, McGrath P, Connolly D, McCluskey DR (2007). Teaching basic life support to school children using medical students and teachers in a “peer-training” model – results of the “ABC for life” programme. Resuscitation.

[23] Whitfield RH, Newcombe RG, Woollard M (2003). Reliability of the Cardiff Test of basic life support and automated external defibrillation version 3.1. Resuscitation.

[24] Plant N, Taylor K (2013). How best to teach CPR to schoolchildren: A systematic review. Resuscitation.

[25] Kelley J, Richman PB, Ewy G, Clark L, Bulloch B, Bobrow BJ (2006). Eight grade students become proficient at CPR and use of an AED following a condensed training programme. Resuscitation.

[26] Colquhoun M (2012). Learning CPR at school – everyone should do it. Resuscitation.

